# FRAME: femtosecond videography for atomic and molecular dynamics

**DOI:** 10.1038/lsa.2017.45

**Published:** 2017-09-22

**Authors:** Andreas Ehn, Joakim Bood, Zheming Li, Edouard Berrocal, Marcus Aldén, Elias Kristensson

**Affiliations:** Division of Combustion Physics, Department of Physics, Lund University, Lund SE-223 63, Sweden

**Keywords:** imaging techniques, ultrafast photonics, ultrafast spectroscopy, videography

## Abstract

Many important scientific questions in physics, chemistry and biology require effective methodologies to spectroscopically probe ultrafast intra- and inter-atomic/molecular dynamics. However, current methods that extend into the femtosecond regime are capable of only point measurements or single-snapshot visualizations and thus lack the capability to perform ultrafast spectroscopic videography of dynamic single events. Here we present a laser-probe-based method that enables two-dimensional videography at ultrafast timescales (femtosecond and shorter) of single, non-repetitive events. The method is based on superimposing a structural code onto the illumination to encrypt a single event, which is then deciphered in a post-processing step. This coding strategy enables laser probing with arbitrary wavelengths/bandwidths to collect signals with indiscriminate spectral information, thus allowing for ultrafast videography with full spectroscopic capability. To demonstrate the high temporal resolution of our method, we present videography of light propagation with record high 200 femtosecond temporal resolution. The method is widely applicable for studying a multitude of dynamical processes in physics, chemistry and biology over a wide range of time scales. Because the minimum frame separation (temporal resolution) is dictated by only the laser pulse duration, attosecond-laser technology may further increase video rates by several orders of magnitude.

## Introduction

The ability to spectroscopically probe ultrafast events has been of vital importance for understanding fundamental scientific questions in biology, chemistry and physics^1, 2, 3, 4, 5, 6, 7, 8, 9, 10, 11^. Examples of such investigations include charge–charge interactions dynamics^12^, transition-state dynamics of chemical reactions^7^ and the photosynthetic process^13^. Traditionally, such phenomena have been studied with pump/probe arrangements, in which the time delay between the pump and probe are scanned sequentially; however, such methods are limited to statistical studies under reproducible conditions. Understanding ultrafast dynamic events under more arbitrary conditions, however, requires methods that can acquire a sequence of temporally and spatially resolved data from a single ultrafast event. Apart from the requirement of extremely high temporal resolution, such methods should ideally also be able to pump/probe and analyze light of arbitrary wavelengths. Although current state-of-the-art ultrafast video-sequence two-dimensional (2D) imaging methods are capable of capturing single events with frame rates up to 1 THz^14^, they lack the ability to illuminate samples at arbitrary wavelengths and/or to detect spectrally complex signals, thus preventing these methods from being compatible with most spectroscopic approaches to extract species-specific information^15^ or quantum state dynamics^2^.

Previously described methods for achieving rapid data acquisition have relied on either detectors with extremely high temporal resolution or schemes based on ultra-short laser probes. For example, recent advances in tomographic reconstruction^16^ and transillumination shadowgraphic imaging^14^ use the relatively large spectral bandwidth offered by femtosecond (fs) laser pulses for spatiotemporal distinction. However, although these probe-based methods^14, 16^ are able to achieve ultrafast video rates, their methodologies are based on spectrally dispersive detection, thus preventing the signal from having arbitrary spectral characteristics. Sequentially timed all-optical mapping photography (STAMP)^14^, which can provide video rates as low as 1 picosecond (ps), relies on probe pulses with distinctly separated wavelengths, thereby excluding unification with most spectroscopic techniques. The methodology presented by Li *et al.*^16^ also relies on a spectral separation detection scheme. However, that work was based on tomographic imaging, thus enabling three-dimensionally resolved videography.

An alternative videography method with potential for spectroscopic compatibility is the use of ultrafast detectors that temporally resolve signals. Such a detection scheme has been demonstrated by Gao *et al.*^17^, who have combined a streak camera^18^ and compressed ultrafast photography (CUP). In contrast to probe-based methods, CUP has significantly decreased (hardware-limited) temporal resolution of just above 30 ps^17^. In addition, streak cameras suffer from poor spatial resolution and quantum efficiency—crucial aspects for compressive imaging to avoid image artifacts^19^. Hence, no previously described state-of-the-art methods for 2D videography of dynamic single events are able to unify femtosecond temporal resolution with spectroscopic compatibility.

In this article, we present a laser probe-based method that unifies the aforementioned aspects and is capable of producing a video sequence of non-repetitive dynamic events at femtosecond timescales and beyond for spectroscopy. Our method, called Frequency Recognition Algorithm for Multiple Exposures (FRAME), allows acquisition of a series of laser-induced images at frame rates that are limited only by the laser pulse duration. Here we used femtosecond laser pulses to demonstrate videography with record high sub-ps (THz) temporal resolution, although attosecond laser pulses could, in principle, increase the frame rate into the PHz regime. The novelty of the FRAME concept lies in superimposing a structural code onto the illumination to encrypt a single event, which is then deciphered in the data post processing. Because each image in the video sequence is extracted by using a unique spatial code, the method does not rely on a specific optical wavelength or laser bandwidth, and hence can be used for spectroscopic measurements. Here we provide a proof-of-principle demonstration of our method by performing imaging light-in-flight—often regarded as the gold standard experiment for ultrafast imaging^14, 16, 17, 20^ at 5 THz, a timescale on which even light appears stationary on a macroscopic scale.

To further demonstrate the wide applicability and versatility of FRAME, we present spectroscopic videography measurements based on laser-induced fluorescence for resolving fluid dynamics in both liquid- and gas phase, thus illustrating how FRAME can capture motions occurring on vastly different timescales. Our findings demonstrate the potential to perform a wide range of scientific investigations of transient, non-repetitive events by expanding the dimensions of spatiotemporal information. Such capabilities enable (i) new measurement concepts (such as coherence lifetime imaging), (ii) visualization of dynamic processes (such as optical filamentation, plasma formation and photoinduced chemistry in heterogeneous organic compounds), (iii) technical possibilities to circumvent the complications of mechanical vibrations in ultrafast measurements^21^ and (iv) increased statistical sampling provided by cameras rather than single-point detectors.

## Materials and methods

### FRAME methodology

**Figure 1:**
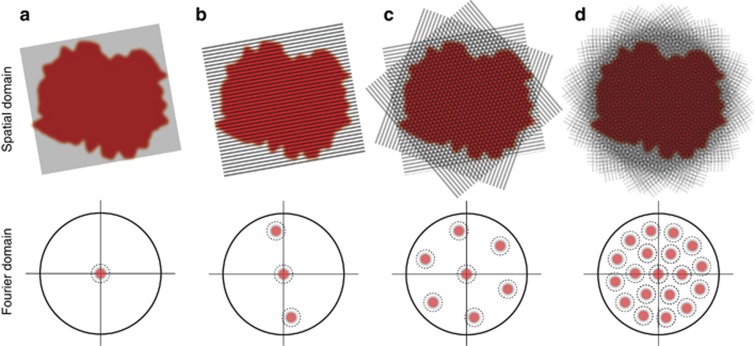
The operating principle of FRAME. (**a**) A uniformly illuminated sample with an image content that resides primarily near the origin in the Fourier domain. The outer circle marks the resolution limit of the detector. (**b**) Illuminating the sample with sinusoidal intensity modulation effectively places two ‘image copies’ of the object structures in the otherwise unexploited space in the Fourier domain. (**c**, **d**) Each ‘image copy’ fills only a fraction of the available reciprocal space, thereby allowing for multiple-illumination schemes without signal cross-talk.

The FRAME methodology is graphically illustrated in Figure 1. The methodology is based on the simple premise (which is analogous to several methods used for digital image compression) that the power spectrum of an image is strongly shifted toward low spatial frequencies^22^. Figure 1a shows a uniformly illuminated sample together with its Fourier transform. Illuminating the same target with a sinusoidal modulation with a spatial frequency of 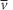 effectively places a pair of ‘image copies’ of its object structures at 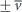 in the Fourier domain (Figure 1b)—an approach often used in microscopy to improve depth resolution^23^ as well as for super-resolution imaging^24^. If instead the spatial frequency and/or the orientation of the modulation are altered for a number of illuminations, each respective ‘image copy’ may be strategically placed at other regions in reciprocal space (Figure 1c and 1d). After data acquisition, each frame (image copy) stored on-chip is accessed individually through a frequency-sensitive 2D spatial lock-in algorithm. In this process, each individual frame is first isolated in reciprocal space by means of a 2D frequency band-pass filter, thus effectively removing all other image copies. The dimensions of this band-pass filter set the final resolution of the extracted frames. The isolated data are then digitally transferred to the origin in the Fourier domain, thus transforming the modulation amplitude into a dc component. Application of an inverse Fourier transform on this filtered and rearranged data set transforms spatial frequencies into intensity values, thus revealing the image information that was stored at an offset location in reciprocal space. More details on the image analysis can be found in Supplementary Information.

From a practical perspective, the FRAME approach allows a single camera to accept a multiple number of 2D signals in one exposure. Fundamental limitations related to high electronic readout speeds that commonly restrict videography are thus circumvented, and consequently low-noise and high-sensitivity imaging sensors with a large dynamic range and high pixel resolution can be used. These features are important because all image copies may need to share pixels and thus also share the full well capacity (dynamic range) of the camera. For example, eight image copies whose spatial structures overlap would each have a 13-bit resolution if captured with a 16-bit sensor.

### Spectroscopic experiment

**Figure 2:**
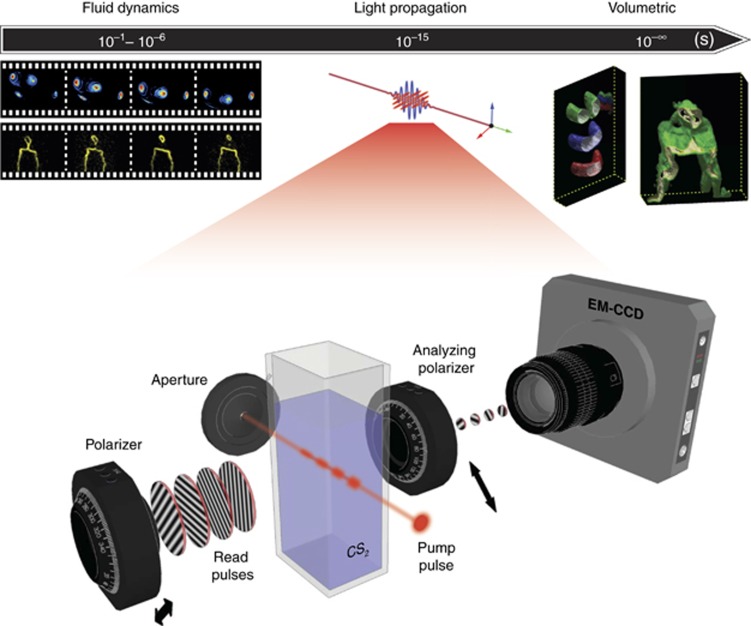
Proof-of-principle demonstrations of FRAME and schematics of the setup for visualizing a femtosecond light pulse. FRAME allows for measurements at vastly different time scales, from spectroscopic studies of fluid dynamic events occurring at second-to-microsecond timescales, through femtosecond videography, to temporally resolved volumetric, four dimensional, realizations, as described in more detail in the Supplementary Information. The lower panel shows the schematics of the setup used to visualize the propagation of a femtosecond light pulse through a Kerr-sensitive medium.

FRAME is adaptable for several optical configurations, such as for pump-probe-, wave mixing-, transmission- and back/side-scattering measurements. To demonstrate this versatility, we conducted four experiments using FRAME. The results demonstrating the spectroscopic capability of FRAME are shown in the insets in Figure 2. These results display planar laser-induced fluorescence videography as well as instantaneous volumetric measurements in liquid and gas. The gas dynamics displays the formaldehyde distribution in a turbulent flame, whereas the movement of a droplet, marked with dye, is captured in a liquid. These experiments were conducted with a side-scattering probing geometry, and experimental details from these measurements are further described in the Supplementary Information.

### Femtosecond videography experiment

Our main proof-of-principle demonstration of FRAME focused on the extreme temporal resolution offered by this approach, which is higher than the resolution of previously reported methods for 2D videography. To demonstrate femtosecond videography, we performed a pump-probe measurement to monitor the propagation of a femtosecond laser pulse through a Kerr-sensitive medium (CS_2_) by recording with a pulse-to-pulse separation between the read (probe) pulses—defined here as temporal resolution—corresponding to a frame rate of up to 5 THz. In the experiment (lower panel in Figure 2), a 125 fs long Ti:Sapphire laser pulse at 800 nm, the pump pulse, was directed through the Kerr medium. A pulse train of four read pulses arrived at the Kerr medium with a direction of propagation orthogonal to the pump pulse. The Kerr gate uses two polarizers, rotated 90° relative to each other, thus preventing the read pulses from reaching the detector. However, when the arrival time for the pump pulse and a read pulse coincide within the Kerr medium, the birefringence caused by the pump pulse allows the overlapping part of the read pulse to be transmitted. The Kerr gate is, however, not binary; instead, its gate function is governed by the intensity profile of the pump beam and is thus superimposed onto the transmitted light. This effect was exploited here to visualize the pump beam as it propagates through the Kerr medium. The intensity profile of each read pulse was encoded with a unique spatial modulation structure, created by imaging the ±1 orders of diffraction of a Ronchi grating (20 lp mm^−1^) into the measurement volume. After exiting the Kerr medium, the transmitted signals were imaged onto a single camera chip (Andor Luca, Andor Technologies, Belfast, UK, 1002 × 1004 pixels).

The burst of read pulses was created by splitting a second output from the Ti:Sapphire laser by means of a beam-splitter arrangement. In this proof-of-concept experiment, the time of arrival for each read pulse was controlled via separate optical delay lines; however, both these and the encoding optics could, in principle, be replaced by a single photorefractive volume reflection hologram^25^. The spatial modulation can be formed by either projecting or imaging the grating into the probe volume. The latter approach (used in the current setup) is beneficial because potential issues caused by color dispersion (diffraction) of the read pulses are avoided. Experimental details are further presented in Supplementary Information.

## Results and discussion

**Figure 3:**
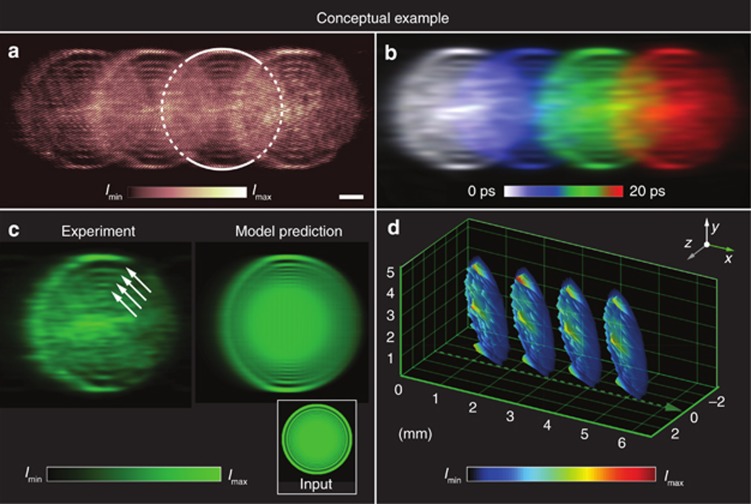
Conceptual example of a FRAME video sequence, imaging the propagation of a light pulse in the setup depicted in Figure 2. (**a**) The raw data as seen by the camera, recorded at 150 GHz. Scale bar=1 mm. The highlighted frame is compared with the model predictions in **c**. (**b**) Video sequence after processing, with individual frames color-coded. (**c**) The third frame of the video sequence extracted using the FRAME algorithm is in good agreement with a model prediction of the expected beam shape. The arrows highlight internal rings that can be identified in the beam profile. (**d**) Geometric representation showing the spatial cross-sections where the pump pulse (propagating in the *x* direction) and the read pulses (propagating in the *z* direction) interact. Note that the camera observes the projection of this signal along the *z* direction.

In Figure 3a, we present a conceptual example of a raw data video sequence that we recorded using the FRAME arrangement operated at relatively low frame rates of 150 GHz (for illustrative purposes). Despite being spatially overlapped, the FRAME algorithm is able to separate each read pulse individually (Figure 3b), thereby revealing structural details in the intensity profile of the pump beam (Figure 3c). The orientation relaxation of CS_2_ molecules is, however, expected to distort the image of the pump-beam profile; the loss of clarity of the diffraction pattern from the front aperture (Figure 3c), manifested as a ring along the rim of the pump-beam profile, may be an indication of this effect. Notably, the relaxation time of the Kerr effect cannot be separated from the motion of the pump pulse in the current data. We constructed a 3D model of the measurement to verify that this observation was caused by the relaxation of the Kerr effect; our experimental data were in good agreement with the model predictions (Figure 3c). Figure 3d displays a spatial representation of where (in space) the pump and read pulses interact (geometrical calculation based on a constant speed of light). Further illustrations of how the detected signal is created as the pulses interact in the probe volume are presented in the Supplementary Information.

The operation of FRAME does not inherently generate any beam dispersion and thus preserves the entire bandwidth of the laser. This characteristic allows FRAME to take full advantage of the short laser pulse duration to maximize frame rates and minimize exposure (interaction) times. Under conditions of no temporal overlap between individual frames, the pulse duration dictates the frame rate, and the maximum is achieved when the read pulses are arranged ‘back-to-back’. In the current study, the read pulse duration was ~125 fs, yielding a maximum frame rate of ~8 THz, which is best compared to the ~33 GHz frame rate achievable with CUP^18^ (despite the conceptual differences between the two approaches). However, the temporal resolution of FRAME, in principle, has no upper limit and higher frame rates could potentially be realized by overlapping individual frames in time; however, this approach would not necessarily allow faster events to be traced in practice, because the images in the sequence would not be temporally resolved. In addition, movements or structural changes occurring on a macroscopic level start to become negligible when frame rates of ~10 THz are approached. For example, in CS_2_, light travels ~23 μm in 125 fs^26^, corresponding to a distance of ~3 pixels on the detector chip in the setup used in this current experiment. Hence, although it would practically be possible to observe the propagation of the pump beam at even higher frame rates, the beam would appear stationary with the current spatial resolution. However, spatial resolution could be improved with high-resolution magnifying optics; for example, a microscopic FRAME arrangement could thus directly benefit from higher frame rates. In addition, some response times of quantized systems occur on femtosecond^3, 4, 7^ (or even attosecond^1, 2^) timescales, and tracking such dynamics would therefore require THz frame rates or higher.

**Figure 4:**
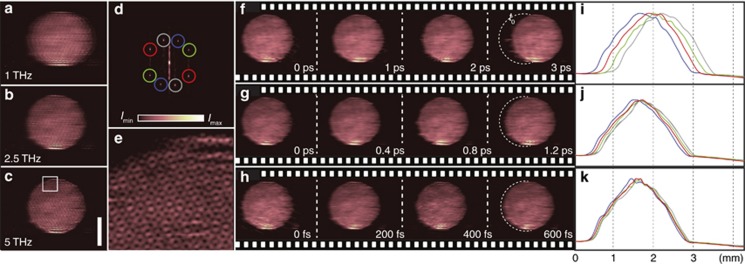
Video sequences with different frame rates are shown. A sequence of four images acquired at a frame rate of (**a**) 1 THz, (**b**) 2.5 THz and (**c**) 5 THz in one image acquisition. Scale bar=2 mm. (**d**) The 2D Fourier transform of **a**, with the image copies from each read pulse marked. (**e**) Magnified view, highlighting the superimposed coded structure. (**f**–**h**) Extracted frames from the video sequence in **a**–**c**. The dashed half circle indicates the first frame. (**i**–**k**) Vertical summations of the four images **f**–**h** showing the pump pulse as it propagates in the CS_2_ liquid.

Next, we demonstrated the temporal capabilities as well as the high image quality and low shot-to-shot fluctuations that can be realized with FRAME. Figure 4 shows three single-shot video sequences of the propagation of the pump pulse through the Kerr medium, which were captured using the FRAME setup at 1, 2.5 and 5 THz. Figure 4a–4c shows the unprocessed data as seen by the camera, and Figure 4d displays the Fourier transform of Figure 4a, wherein the object structures from each read pulse appear as isolated peaks symmetrically around the origin in the center. Most structural information resides near the origin, thus leaving the majority of the Fourier domain otherwise unexploited, with vacant space for even more image copies (Figure 1). An example of an image encoded with 16 frames is presented in Supplementary Fig. S10. Application of the frequency-sensitive algorithm extracts results from each read pulse (Figure 4f–4h). To the best of our knowledge, one-time events have not previously been tracked with such high temporal resolution (200 fs).

**Figure 5:**
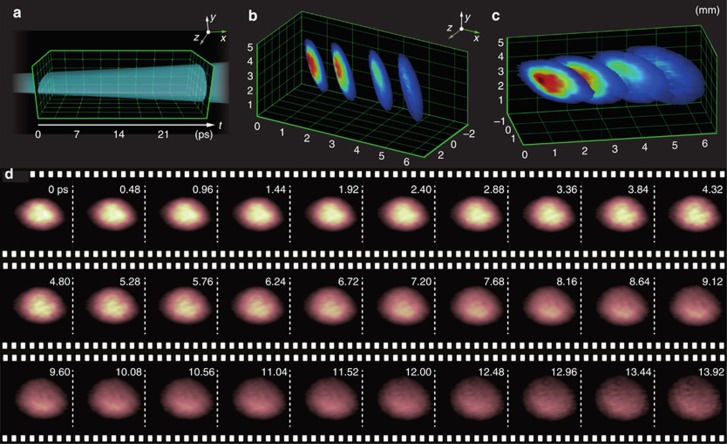
FRAME video sequence of a focused light pulse. (**a**) A 3D view of the probe volume where the propagating laser pulse (pump) is diverging in the *y* axis direction. (**b**, **c**) Different views of the interaction zone where the pump- and read pulses interact as the pump pulse propagates in the medium. (**d**) A reconstructed image sequence of the wave front of the pump pulse as it propagates through the CS_2_ liquid using 3D interpolation. The cross-sectional image plane is perpendicular to the pointing vector of the laser beam (*x* axis), displaying the interpolated beam profile of the diverging pulse.

Finally, we demonstrated the ability to perform videography of a moving target undergoing transformation by using FRAME. Because both the pump- and read pulses propagated as they interacted, each separate frame did not, in fact, constitute a truly instantaneous representation. This ‘spatiotemporal effect’, which is somewhat analogous to motion blur, is not a direct concern for the data presented in Figures 3 and 4, because the beam profile is constant and is conserved throughout the video sequence. However, when visualizing dynamic objects that evolve at or near luminous speed, this unavoidable effect may become apparent. Because FRAME is capable of acquiring several images of a single event, interpolation schemes can be used to estimate the intermediate states of the complex dynamics of such objects. To demonstrate this capability, we used 3D interpolation to reconstruct the wave front of a diverging laser pump pulse as it propagated in the Kerr medium (Figure 5). Figure 5a shows a schematic of the path of the diverging pump beam through the measurement volume, and the spatially distributed data (similar to Figure 3d) are displayed from two different angles in Figure 5b and 5c. The outcome of the interpolation analysis, shown in Figure 5d, revealed the derived time evolution of the wave front from a single laser pulse while traversing the Kerr medium (in a plane perpendicular to the direction of propagation).

The FRAME system (Figures 3–5) used here had a final spatial resolution of ~15 lp mm^−1^ (over a field-of-view of 7 × 7 mm^2^). To improve a FRAME system, that is, by increasing the number of video frames and/or improving the spatial resolution of each frame, the Fourier domain of the detection system first must be expanded. Expanding the Fourier domain can be achieved by (i) increasing the number of pixels of the imaging sensor and (ii) improving the optical resolution accordingly. Such an optimized detection system would allow a larger set of frames, carried by low- and high-modulation frequencies, to be strategically positioned in reciprocal space, as exemplified in Figure 1d. Experimentally, this involves (i) a tailored set of gratings, each with a unique spatial frequency and orientation, (ii) an imaging sensor with a large number of pixels and (iii) a high-resolution optical system. An example of such a system is provided in the Supplementary Information. Notably, if spatial resolution is critical, then the frames should be maximally dispersed in reciprocal space to allow the use of large filter functions in the spatial lock-in algorithm.

## Conclusions

To our knowledge, the reported methodology, FRAME, is the first to enable ultrafast 2D videography having spectroscopic compatibility with both high spatial and temporal resolution, down to at least femtosecond timescales. In contrast to existing image coding techniques^18, 19^ that rely on translation/sweeping solutions, FRAME is entirely light-based; a boost in frame rate by more than two orders of magnitude is demonstrated herein. Because FRAME is restricted to neither a specific wavelength nor any particular optical configuration, it is compatible with most light–matter interaction measurements using standard laboratory equipment, such as those based on absorption, scattering, fluorescence, polarization, wave mixing and coherence. Utilizing imaging sensors with large number of pixels in conjunction with high-resolution imaging optics would increase the image-storing capacity to allow a larger number of frames in the image sequence. Further, FRAME, together with the current progress within ultrafast science toward producing laser pulses in the attosecond regime^27^, could, in principle, offer video rates up to a thousand times faster than those presented herein.

The simple yet unique ability of FRAME to acquire several laser images in one recording also enables new measurement opportunities. For example, combining FRAME with currently existing laser methods for fluorescence lifetime imaging^28^ (FLI) of single events^29^ would allow all FLI data to be collected in one image recording. Finally, the ultimate purpose of single-event videography is to use the existing knowledge of ultrafast dynamics gained from studies of reduced systems to understand evolving global systems; we believe FRAME can facilitate achievement of this endeavor.

## Author contributions

AE, EK, JB and MA designed the research. AE set up the experiment, and EK and AE performed the data collection. EB provided experimental insights and EK performed the data analysis. ZL and EK performed the flame measurements. EK and AE wrote the paper, with extensive suggestions from all other co-authors.

## Supplementary Material

Supplementary Matreial
