# Supplementary material for: FRAME: femtosecond videography for atomic and molecular dynamics
Source: Light Sci Appl. 2017 Sep 22;6(9):e17045–. doi: 10.1038/lsa.2017.45 (PMC6062331; doi:10.1038/lsa.2017.45)
Supplement: Supplementary Matreial [file lsa201745x1.pdf]

FRAME – femtosecond videography for atomic and molecular dynamics

Authors: Andreas Ehn, Joakim Bood, Zheming Li, Edouard Berrocal, Marcus Aldén*

& Elias Kristensson

Division of Combustion Physics, Lund University, Sweden

*Corresponding author: Prof. Marcus Aldén

Division of Combustion Physics, Lund University

Professorsgatan 1, SE-223 63, Lund, Sweden

Fax: + 46 46 222 45 42

Phone: + 46 46 222 76 57

Email: marcus.alden@forbrf.lth.se

1. Experimental details of the time-of-flight experiment

Figure S1 shows the optical arrangement that is employed in the main experiment. The laser resonator generates two synchronized laser pulses with a wavelength and pulse duration of 800 nm and 125 fs, respectively. The first pulse is used as a pump pulse to activate the Kerr medium in the probe volume. The second output is split into four equally intense beams using a beam splitter configuration. Each beam is guided into an optical delay line to control the arrival time of each pulse separately. After being delayed, the beams are sent through individual Ronchi gratings (20 lp/mm), each with a unique orientation. The four beam lines are then overlapped with a second beam-splitter arrangement. The ±1 diffraction orders of the light is then collected with an imaging lens (f=150 mm, ∅=50 mm) that forms sinusoidal fringe patterns in the probe volume (similar to [24]). This requires that the distance between the lens and the gratings are the same for all four beam lines. Two crossed polarizers on each side of the Kerr medium (which is the probe volume) form a Kerr-gate setup. Such a setup will only transmit light through the Kerr gate when the probe and read pulses intersects in the Kerr medium. The transmitted light, in turn, will be imaged onto the camera sensor, which is situated directly after the analyzing polarizer.

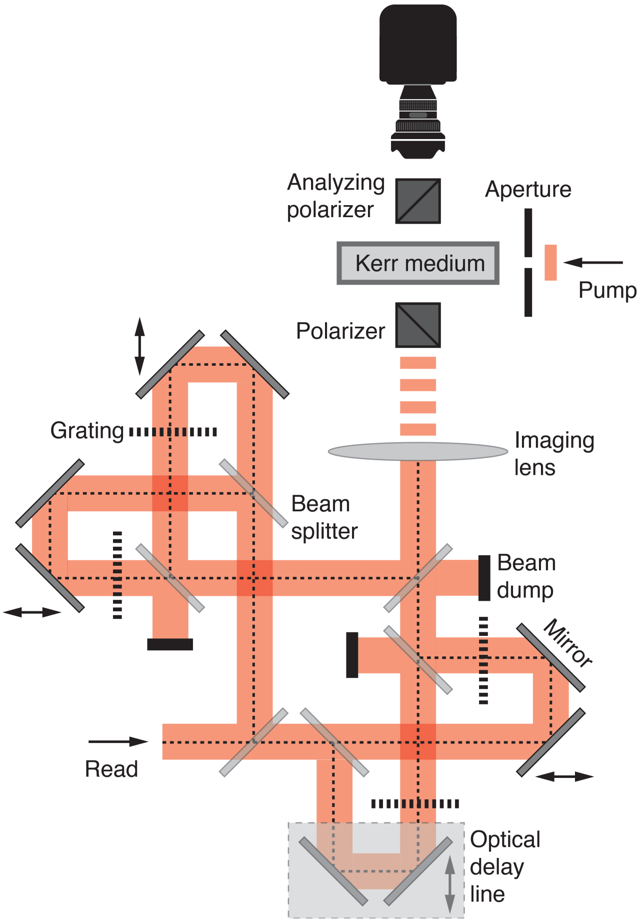

Figure S1. Experimental setup overview. The laser resonator offers two synchronized 125 fs long laser pulses (800 nm), each with a pulse energy around 27 mJ. The read pulse was split up into four different beam lines using beam splitters. Each beam was then guided through uniquely orientated Ronchi rulings (20 lp/mm), effectively creating the intensity modulation needed for FRAME. After rearranging the four beams collinearly using beam splitters, a positive spherical lens was used to form an image of the Ronchi patterns in the Kerr medium. The time of arrival for each read pulse was adjusted using optical delay lines. The light that is being transmitted through the Kerr gate is imaged into a camera sensor chip.

2. The model of the optical Kerr gate

A 3D model of the optical Kerr gate was constructed to verify our observations in the main experiment. The model simulates how a pump beam, with a given spatial intensity profile propagates in discrete steps through a Kerr-sensitive medium, where the relaxation time of the molecules in the medium can be altered. In the model a read pulse intersects the pump pulse (see Figure S2a-b) and a signal that is dictated by the magnitude of the local birefringence is generated. The projection of this signal (along the direction of propagation of the read pulse) is then imaged onto a camera. To investigate whether the results acquired by our FRAME system is in agreement with the model prediction, we construct an input beam (pump) having a spatial intensity profile according to Figure S2c – representing the diffraction pattern caused by the front aperture. According to the literature, CS_2 has a relaxation time of 2.1 ps, which also gives the best agreement with our experimental data (compare Figure S2f and S2h). See also Supplementary Movie 1, which illustrates how the signal in our model is created as the four read pulses intersect the probe pulse as well as how the projection of this signal is imaged onto the camera. All calculations in the model are based on a constant speed of light.

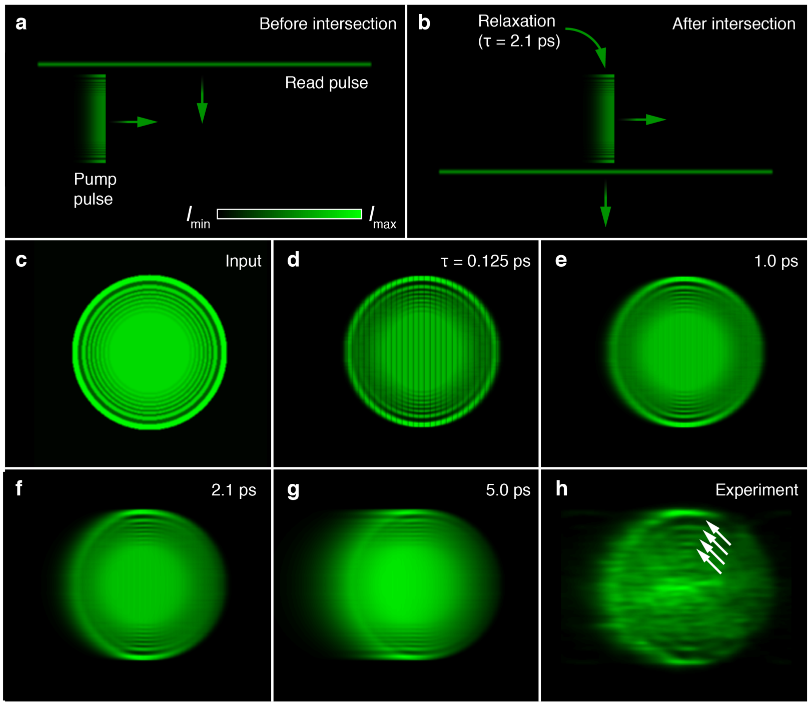

Figure S2. The model of the optical Kerr gate. Top view of the experiment, showing one read pulse and the pump pulse the instance before (**a**) and after (**b**) they intersect inside the Kerr medium. The relaxation time of CS_2 is modeled by exponentially decreasing the magnitude of the birefringence. (**c**) Beam profile of pump pulse, used as input data for the model. The outer ring is generated by diffraction from the front aperture (see Figure 2), whereas the inner, weaker rings are intrinsic features of the laser source. (**d**-**g**) Output of the model for various relaxation times. The experimental data (**h**) shows good agreement with the model predictions (case **f**).

3. Color dispersion

The optical arrangement of the current FRAME setup (Figure S1) employs transmission Ronchi gratings to generate the intensity modulation for each probe pulse. For broadband laser illumination, this approach thus generates color dispersion due to diffraction according to:

$$\sin\theta_g = \frac{m\lambda}{d} \tag{1}$$

where *m* is the diffraction order, λ the wavelength of the light and *d* the fringe separation of the grating. Figure S3 illustrates the phenomenon for *m*=1. For an ultrashort, broadband laser pulse, color dispersion thus leads to temporal differences between the color constituents of the pulse, where the shorter wavelengths arrive first – an undesired condition. To solve this potential issue, the FRAME setup is based on *imaging* rather than *projection*, so that the chirp induced by the gratings are compensated for optically (see Figure S3). Mirrors instead of lenses should be used in case of shorter pulse durations to avoid shirping of the laser pulses.

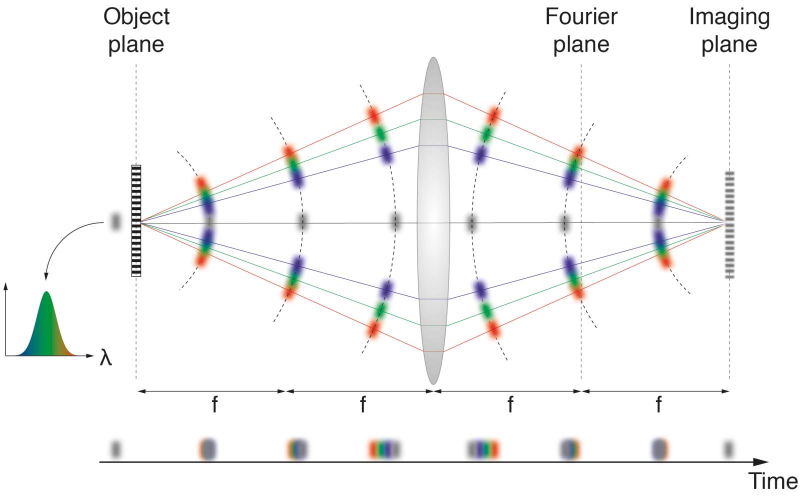

Figure S3. Color dispersion. A broadband laser pulse becomes spectrally dispersed when transmitted through a Ronchi grating, effectively delaying longer wavelengths relative to shorter ones. To avoid this issue, the FRAME setup presented in this paper is based on *imaging* rather than *projection*, so that all color constituents arrive at the same time in the probe volume. It is thus essential to use achromatic lenses or concave (imaging) mirrors when broadband lasers are employed.

4. Spectroscopic measurements

To demonstrate the versatility of FRAME, we have conducted several experiments additional to the light-in-flight demonstration being presented in the main manuscript. The spectroscopic capability of FRAME is demonstrated in Figures S4-S6 using a side-scattering configuration for laser-induced fluorescence in liquids and gases. The experimental arrangement used in these particular experiments is shown in Figure S7-S8. Specifically, we studied the formaldehyde distribution in a turbulent flame at video rates of 10 kHz, capturing the creation of a flame island (Figure S4). In Figures S5-S6 we show laser-induced planar videography of liquids as well as temporally resolved instantaneous volumetric measurements to demonstrate the adaptability of FRAME for alternative optical configurations.

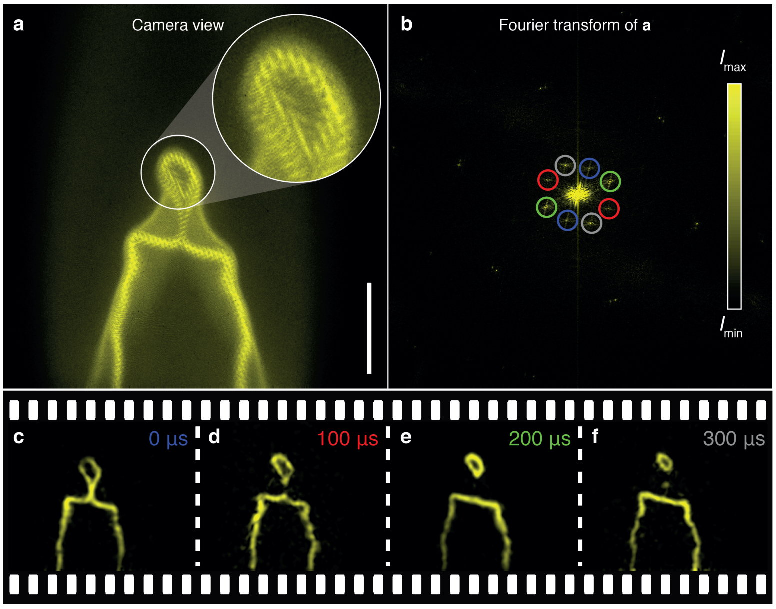

Figure S4. A FRAME measurement for species-specific planar laser-induced fluorescence imaging. A turbulent flame was probed at four instances using differently coded laser sheets, all tuned to stimulate emission from the formaldehyde molecule. (**a**) The raw, unprocessed data video sequence, as seen by the camera positioned for 90 degrees detection. (**b**) The Fourier transform of (**a**), where the locations of the four image copies are marked in different colors. (**c-f**) Video sequence extracted by the FRAME algorithm, showing the creation of a flame island. Scale bar: 5 mm.

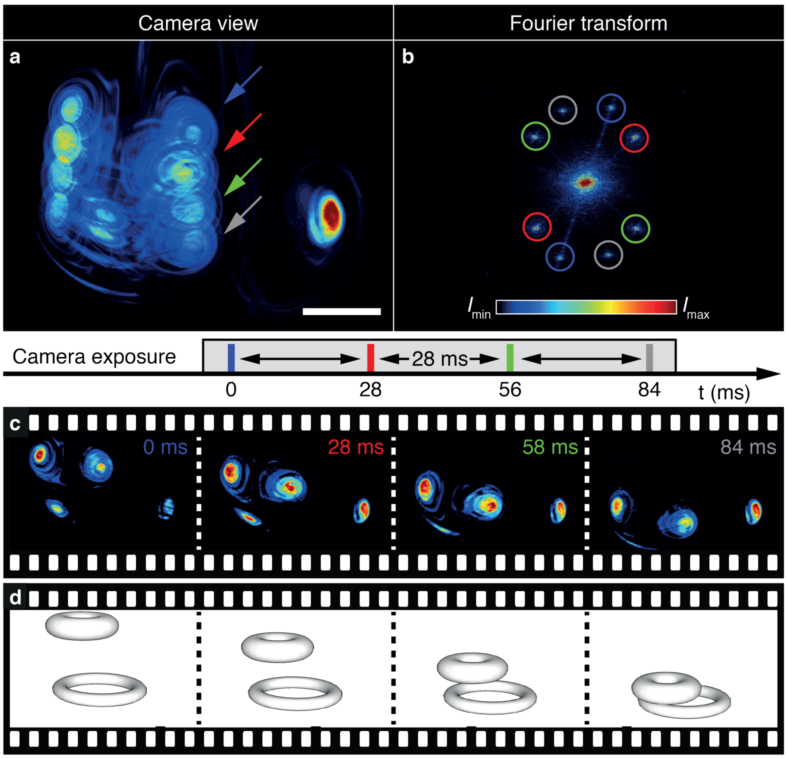

Figure S5. A FRAME experiment using a laser sheet configuration for laser-induced fluorescence of a liquid (two droplets falling). (**a**) A raw, unprocessed video sequence, as seen by the camera and (**b**) its Fourier transform. In the example, four laser sheets probed droplets of dye (shaped like donuts) falling through water, successively. The time in between each illumination event was 28 ms and the arrows in (**a**) indicate the downward progression. (**c**) Analysis of the acquired sequence reveals an interaction between two droplets, where a slower-moving droplet is caught up by a faster. (**d**) A schematic illustration of the event. Scale bar: 5 mm.

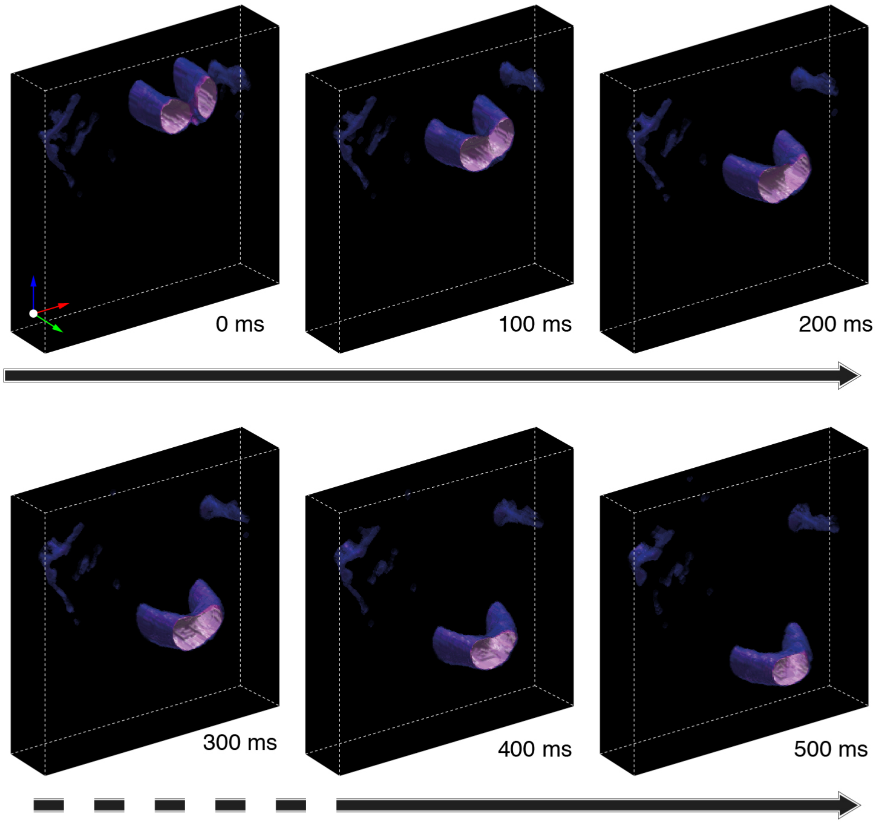

Figure S6. Temporally resolved 3D imaging using FRAME combined with laser sheet illumination. Laser sheet scanning is a common approach to acquire volumetric data. By combining FRAME with laser sheet illumination, where each laser sheet is coded differently, the time-consuming layer-by-layer scanning procedure can be circumvented. The recorded 3D sequence shows a droplet of dye falling through a cuvette with water. Every instance is acquired using four differently coded laser sheets, each probing a different layer. The camera collects the signal from all layers simultaneously and extracts them in the data post-processing using the FRAME algorithm.

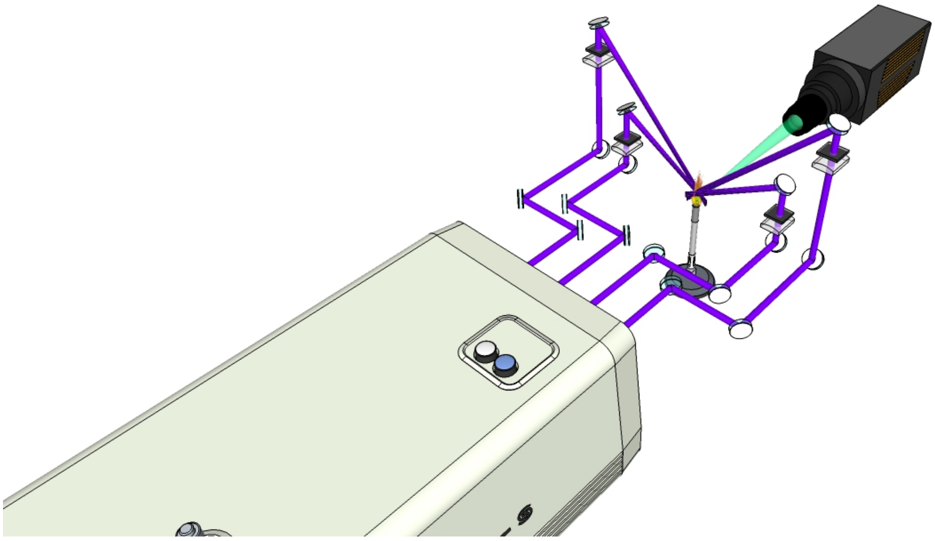

Figure S7. Optical arrangement for the spectroscopic- and laser-sheet experiments using FRAME. Four laser pulses at 355 nm were each shaped into intensity modulated laser sheets, arriving at the sample with different angles to shift each image information to different regions in the Fourier domain.

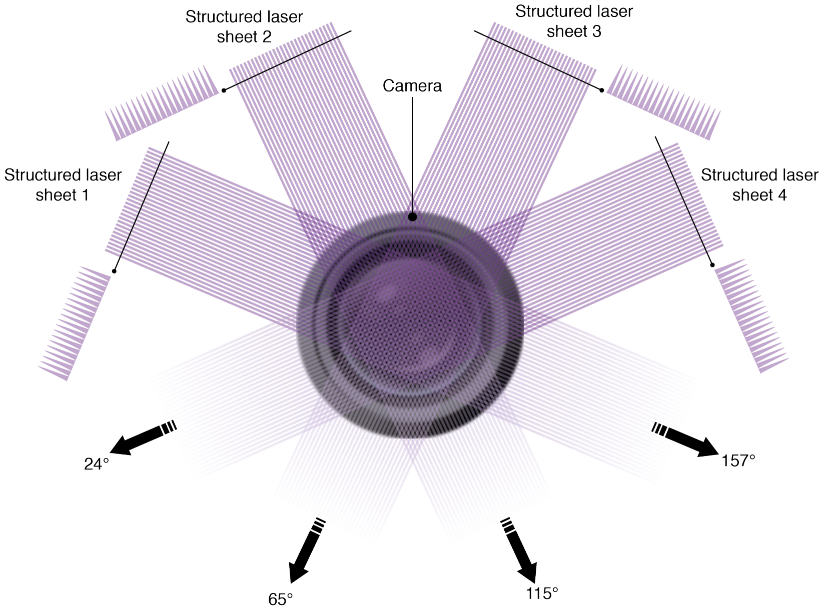

Figure S8. Detection arrangement for the laser sheet experiments using FRAME. The four intensity-modulated laser sheets arrive at the region of interest from different angles (24°, 65°, 115° and 157°). The cross-sections show the orientation of the intensity modulation relative to the propagation of the laser sheet.

5. Spatial lock-in algorithm

The principle of the spatial lock-in algorithm that is used in FRAME is graphically illustrated in Figure S9. The camera observes the sum of all intensity-modulated images (Figure S9a). In the spatial domain, the information from each event overlap, yet in reciprocal space the data from each modulation is separated from each other (Figure S9b). To access each spatially modulated data individually the spatial lock-in algorithm isolates them one-by-one using a band-pass filter. The filter-function and the filtered data are displayed in Figure S8c and d, respectively. The isolated data needs to be demodulated, which is accomplished by rearranging the Fourier domain, so that the isolated data is transferred to the origin (Figure S9e). This process effectively demodulates the information, turning an ac-component into dc (Figure S9f). This is similar to what is achieved in temporal lock-in where the dc-component is multiplied with the reference signal.

The spatial resolution of the extracted frame depends on the bandwidth of the band-pass filter, the larger the more spatial frequencies it preserves. However, if the bandwidth of the band-pass filter is set too high, cross-talk between neighboring frames starts to appear. Figure S10 displays this relationship between specificity (no cross-talk) and image resolution, where the image from another laser pulse appears in the extracted data when the bandwidth of the band-pass filter becomes too large.

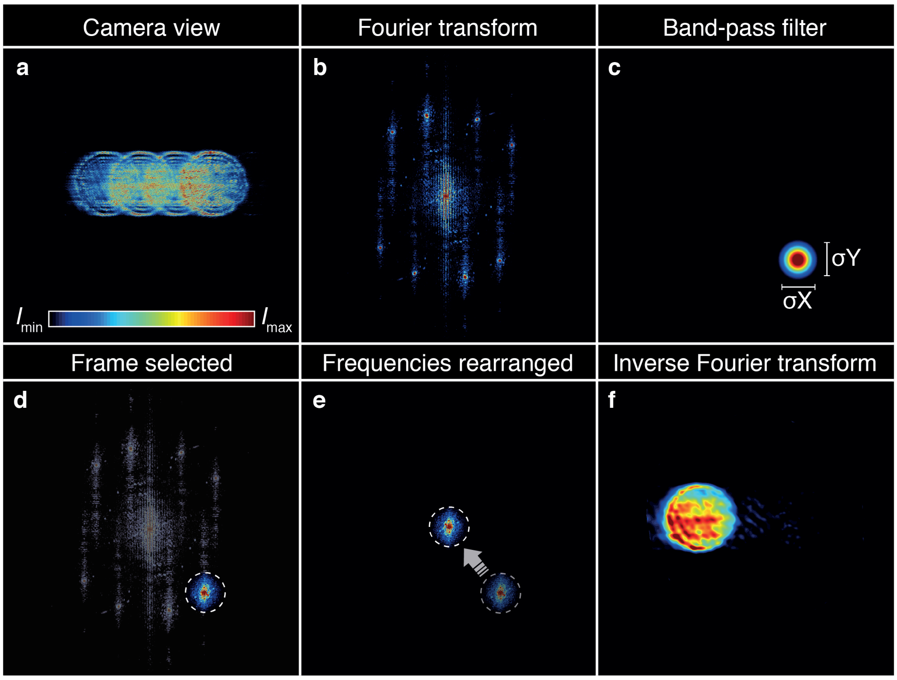

Figure S9. Explanation of the FRAME algorithm. (**a**) A raw data video sequence, as seen by camera and (**b**) its Fourier transform. To access each frame individually, a 2D band-pass filter (**c**), having a super-Gaussian shape, is multiplied with the Fourier transform matrix, which temporarily removes the other image copies being superimposed on the photograph (**d**). (**e**) The filtered data is then digitally transferred to the center of the Fourier domain, which turns a modulation amplitude into a dc-component. (**f**) Applying the inverse Fourier transform of this data reveals the information stored at the offset location.

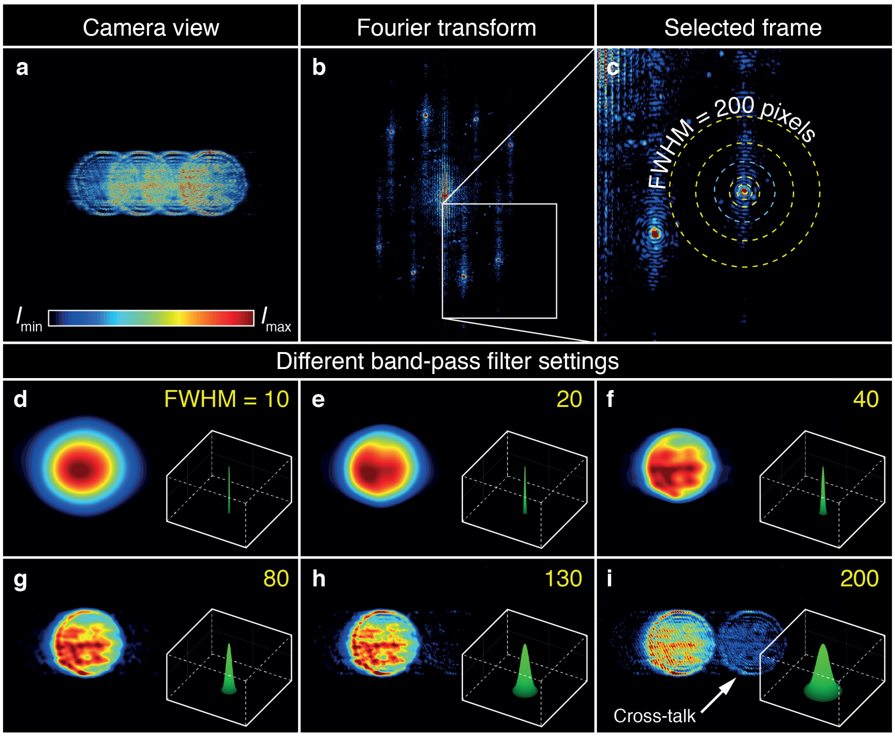

Figure S10. The influence of the band-pass filter. (**a**) A raw data video sequence, as seen by camera and (**b**) its Fourier transform. In the data extraction process, each frame (seen as isolated peaks in (**b**)) is selected using a 2D super-Gaussian band-pass filter. The full-width at half maximum (FWHM) of this filter in pixels (**c**) determines the end resolution of the frames – the larger its kernel, the more frequency content it preserves. (**d-i**) Second frame in the image sequence extracted using different kernel sizes. Small filters provide best selectivity with insignificant interference between neighboring frames, whereas large filters provide best image resolution. The inset shows the band-pass filter.

6. Image-storing capacity (bandwidth) and spatial resolution

The image-storing capacity of FRAME, i.e. the number of frames the camera sensor can store simultaneously on chip – is dictated by (1) the frequency components of the probed object and (2) the spatial resolution of the imaging system. Hence, in terms of image bandwidth, FRAME benefits from high-resolution imaging systems. Figure S11 illustrates how a video sequence with 16 frames can be captured using a high-resolution sensor (4 megapixel). To mimic a real measurement situation, the example is based on the simulated data, seen in Figure S2f. Different strategies can be employed to exploit the space available in the Fourier domain. In the presented experiments in this paper, each intensity-modulated pulse was given the same spatial frequency, while the orientations of the modulations were different. In the example in Figure S11 we have altered both the spatial frequency of the modulation as well as its orientation (see Figure S11c), to better exploit the full capacity of the sensor. When the bandwidth of a FRAME system is exceeded, cross-talk between signal components (see Figure S10i) is likely to occur.

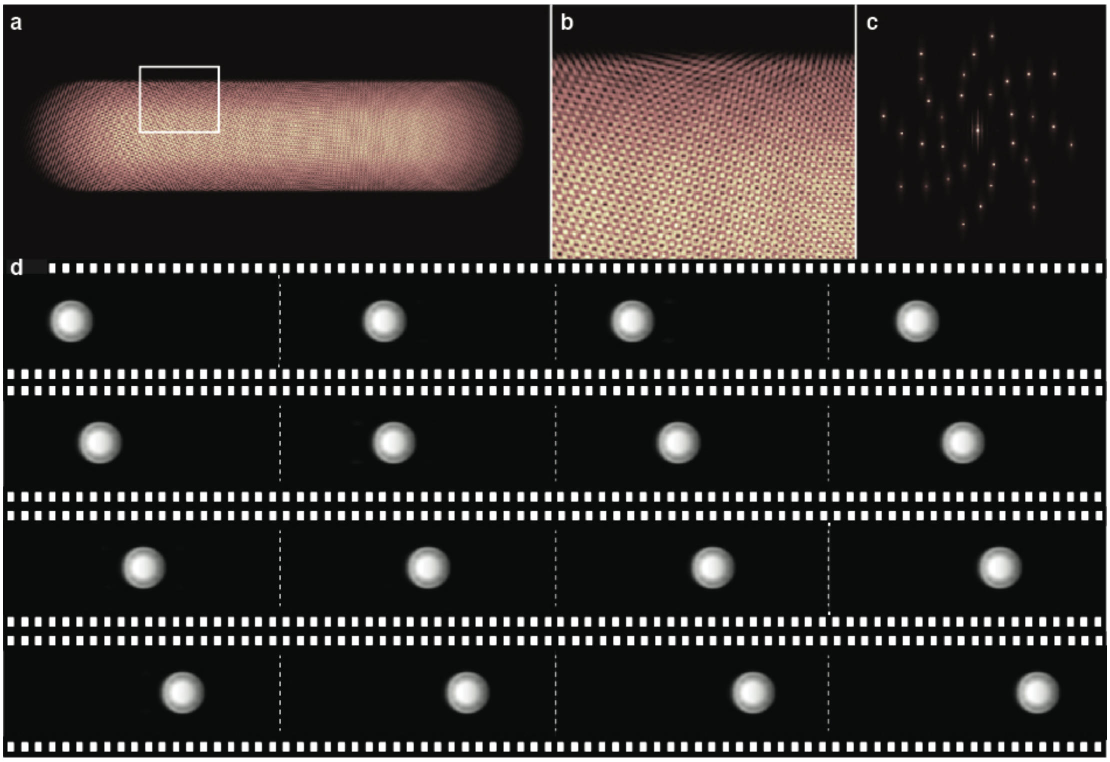

Figure S11. Simulation of the image-storing capacity of FRAME. (**a**) A computer-simulated measurement of a 4 megapixel camera, using the model prediction in Figure S2(f) as input, including a total of 16 individual frames. (**b**) Magnified region, showing the complex cross-pattern seen by the camera. (**c**) Fourier transform of (**a**), showing the strategy to position frames with minimum risk for signal cross-talk. (**d**) Extracted frames from (**a**), revealing the propagation of the laser pulse.

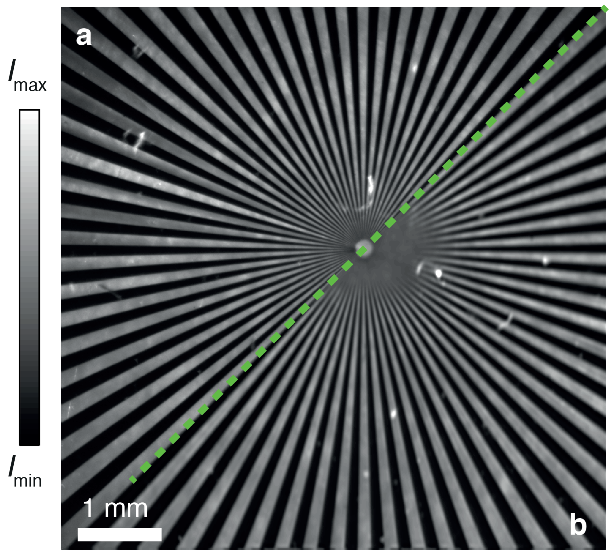

Figure S12. Measurement of the spatial resolution of the current FRAME system. (**a**) Image of a resolution target at full camera resolution, while in (**b**) the image has been filtered using the filter kernel used to extract the individual frames in Figure 4. The result shows how most of the spatial frequencies are preserved in the FRAME data post-processing.

FRAME trades spatial resolution for the ability to acquire several images simultaneously. To investigate this loss in spatial resolution an image of a resolution target was acquired with the FRAME system that was employed in the main experiment. Figure S12a shows the target with no loss in spatial resolution, while Figure S12b shows the same image after being processed with the FRAME algorithm. By analyzing the data, the spatial resolution was found to be 13 lp/mm and 8 lp/mm for the two cases, respectively. Note that the current system employed a detector with a pixel resolution of 1002×1004 pixels, in contrast to the simulation in Figure S11, which was based on 2000×2000 pixels.
